# Periprosthetic femoral fractures in Total Hip Arthroplasty (THA): a comparison between osteosynthesis and revision in a retrospective cohort study

**DOI:** 10.1186/s12891-022-05159-2

**Published:** 2022-03-03

**Authors:** Gianluca Scalici, Debora Boncinelli, Luigi Zanna, Roberto Buzzi, Laura Antonucci, Fabrizio Di Maida, Pietro De Biase

**Affiliations:** 1 Traumatology and General Orthopedics Department, Careggi University Hospital, Largo Brambilla 3, 50100 Florence, Italy; 2 Physical Medicine and Rehabilitation Section, Careggi University Hospital, 50100 Florence, Italy; 3 Department of Experimental and Clinical Medicine, Careggi University Hospital, 50100 Florence, Italy

**Keywords:** Periprosthetic fractures, Hip arthroplasty, Revision arthroplasty, CIRS score

## Abstract

**Background:**

Periprosthetic femoral fractures are challenging complications of hip arthroplasty. They are supposed to be a rare complication, but their incidence is rapidly increasing. Surgical treatment aims to achieve early mobilization and avoid the complications of prolonged bed rest. Aim of this study is to evaluate the clinical outcomes of surgical treatment comparing two surgical approaches: revision arthroplasty (RA) versus open reduction and internal fixation (ORIF).

**Methods:**

Authors retrospectively reviewed a series of 117 patients with total hip arthroplasty treated for periprosthetic femur fractures in the period between January 2013 and March 2018 at a single tertiary referral center. Of these, 70 patients satisfied strict inclusion criteria. Patients were classified according to the Unified Classification System (UCS) and distributed in two groups according to surgical treatment. Clinical outcomes were assessed using the Oxford Hip recorded preoperatively and post operatively, Barthel Score, CIRS score (Cumulative illness rating scale), type of fracture and post-operative complications with a minimum follow up of 1 year.

**Results:**

Nominal univariate statistical analysis revealed significant differences between the post and pre-operative Oxford Hip Score (Δ Oxford) and the surgical treatment (*p* = 0.008) and CIRS score (*p* = 0.048). Moreover, we observed a significant relationship between type of treatment and type of fracture (*p* = 0.0001). Multivariate analyses revealed that CIRS score was independently associated with Oxford Score improvement after surgery (*p* = 0.024).

**Conclusions:**

Data from this case series confirmed that surgical treatment was correlated to type of fracture, according to UCS classification. Patients treated by RA had a better functional outcome than patients treated with ORIF, but these results are strongly influenced from the patients’ age, Barthel index and CIRS score. Also, authors found a correlation between functional outcome and comorbidities evaluated by CIRS score. Based on these data we suggest a multimodal approach to these patients, like those used for proximal femoral fractures.

## Background

**Fig. 1:**
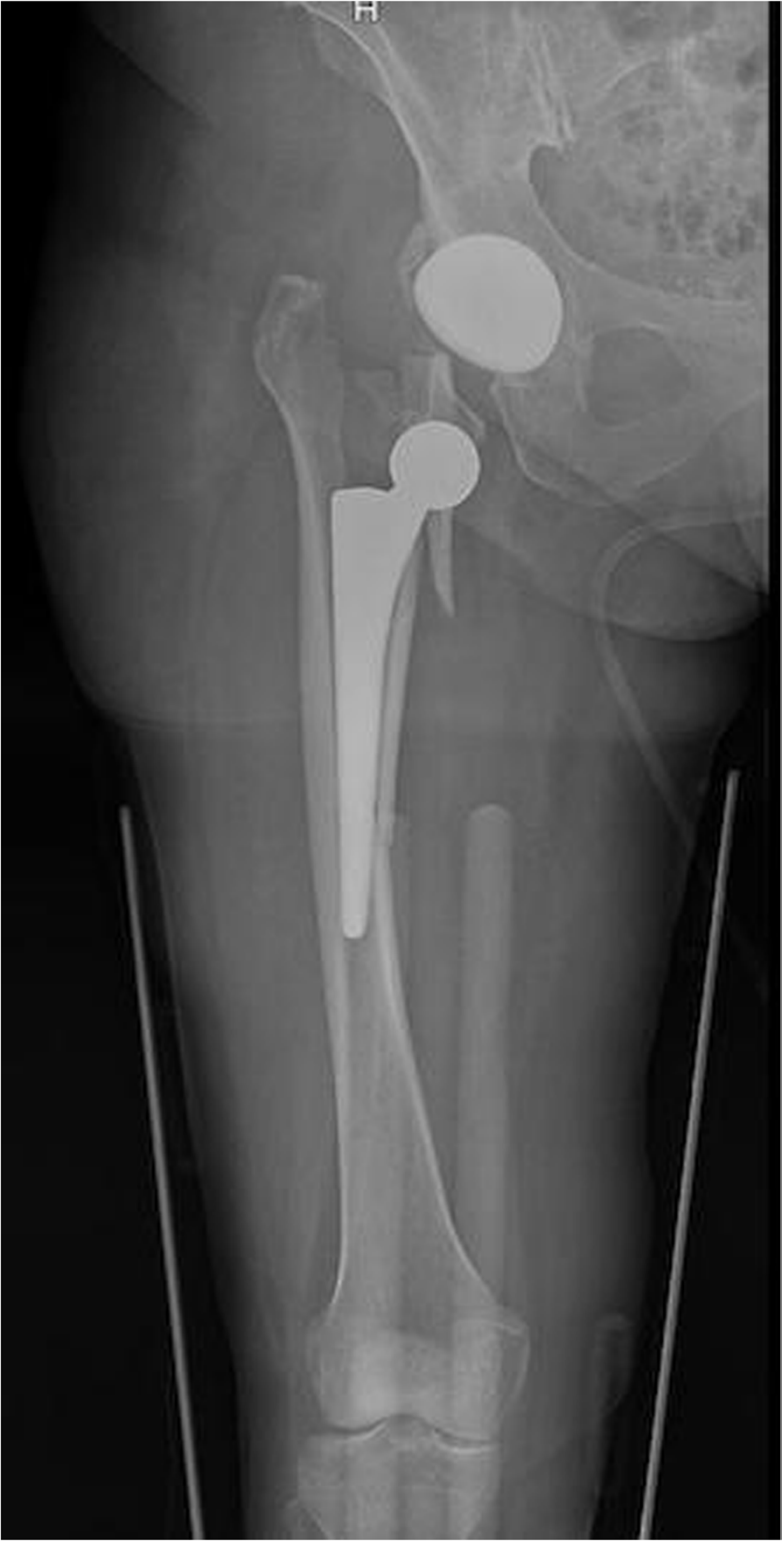
Periprosthetic femoral fracture with stem mobilization

Periprosthetic femoral fractures (PPFx) are challenging complications of hip arthroplasty for orthopedic surgeons and their prevalence is rising. Their incidence is due to an increasing number of prosthetic replacements done every year and higher life expectancy of elderly people with prostheses [1]. According to the literature and Annual Report of the National Joint Replacement Registry (Australian Orthopedic Association) of 2019, there has been a 124.9% increase in primary total conventional hip replacement procedures performed since 2003. Periprosthetic femoral fracture is among the most common causes of revision of primary total hip arthroplasty (20.7%), after loosening (24.6%) and quite the same of dislocation (20.8%) [2]. These numbers consider both immediate perioperative fractures and late postoperative events. However, literature from referral centers reports a lower incidence, with the rate of postoperative periprosthetic at 0.4% in primary stems and 2.1% in revision stems [3, 4]. PPFx involve a significant amount of operating time, bed and cost days and are associated with both high mortality and morbidity [5, 6] (Fig. 1). These fractures are typical of elderly patients, caused by a low energy trauma like a fall from sitting or standing position or a “spontaneous” fracture, usually caused by bone osteolysis or loose prosthesis. The incidence of major trauma is exceptional [7].

Surgical treatments are planned to achieve early mobilization and quick return to basic activities of daily living (BADL) or instrumental activities of daily livings (IADL) avoiding the complications of prolonged bed rest. Obtaining a stable fixation is demanding due to the interference of the femoral stem with synthesis devices and poor bone quality [8, 9]

It has been reported that undergoing revision surgery for periprosthetic fractures results in worse survival outcomes than the general population within 1 year, and this negative influence extends for several years after the surgery [10]. Non-operative management is usually not appropriate for the well-known complications produced by immobilization and for the high percentages of non-unions in fractures around a cemented stem [11]. Surgical treatment depends on fracture site, stem fixation, presence of cement interface in cemented stem and quality of bone around prosthesis. The Unified Classification System (UCS) for PPFx, which is based on the Vancouver Classification relating to femoral fractures as described by Duncan and Masri in 1995 [12], was the one employed in the present study. Classification of PPFx was done on plain radiographs and preoperative CT scans, and it was conformed during surgery with intraoperative evaluation of stem stability. Classification wasn’t the only consideration to decide surgical treatment. Since PPFx are more common in elderly patients, Authors also evaluated comorbidities, preoperative functional status and age of the patient.

The aim of this retrospective study is to validate the use UCS classification in deciding surgical treatment and to evaluate the outcomes of ORIF compared to RA related to functional preoperative status and comorbidities of elderly patients.

## Methods

The Authors retrospectively reviewed a series of 117 patients who had total hip arthroplasty treated for PPFx between January 2013 and March 2018 at our hospital, a tertiary referral center for both trauma and arthroplasty surgeries. All cases were treated by one senior surgeon with more than 10 years’ experience in both RA and ORIF of pelvis and lower limb fractures.

The inclusion criteria for this study were the presence of a periprosthetic fracture around a total or partial hip arthroplasty, the presence of complete clinical and radiological data both in the preoperative and postoperative period. Exclusion criteria were a non-operative treatment due to severe comorbidity and high operative risk, a follow up less than 12 months, pathological fractures for tumors or infection, incomplete clinical or radiological data, like in patients referred from other hospitals. From 117 cases we enrolled 68 patients, accounting for 70 cases, because two patients had bilateral PPFx.

Clinical and demographic data (gender, age, body mass index, side of fracture) were retrospectively gathered from digital clinical records. The highlighted data, the model of prosthetic stem used for revision, the osteosynthesis devices and blood transfusions were collected from (digitally stored) clinical records.

Patients were divided into two groups depending on the surgical treatment, ORIF and RA respectively. Clinical features and overall postoperative complications (infections, hardware failures, dislocations, non-unions, deep venous thrombosis, heart failures and pneumonia) were recorded and related to the fracture classification and the type of surgical management (ORIF or RA). The patients were evaluated for a minimum of four follow-up: 1 month, 3 months, 6 months and 1 year after the surgery. The average time of follow up is 67.2 months.

The periprosthetic femoral fractures were classified and evaluated according to the Unified Classification System (UCS) based on preoperative radiographic, even if the definitive evaluation of stem’s stability was confirmed during the surgical procedures. Fractures are classified as follows: type A, *apophyseal* or extraarticular fracture (A1: avulsion of great trochanter, A2: avulsion of lesser trochanter); type B, *bed* of implant (B1: prosthesis stable and good bone; B2: prosthesis loose, good bone; B3: prosthesis loose and poor bone or bone defect); C, *clear of* or distant to the implant; D, *dividing* the bone between two implants; E *each* of two bones supporting one arthroplasty; F, *facing* and articulating with a hemiarthroplasty [13].

Moreover, we used the CIRS score (Cumulative illness rating scale) to assess patients’ comorbidity level before surgery. This scoring system measures the chronic medical illness taking into consideration the severity of chronic disease across 14 items. Each item has a 0–4 score, where 0 represents “no problem affecting that system” and 4 “extremely severe problem and/or immediate treatment required and /or organ failure and/or severe functional impairment”. The cumulative final score can vary from 0 to 56 [14].

**Fig. 2:**
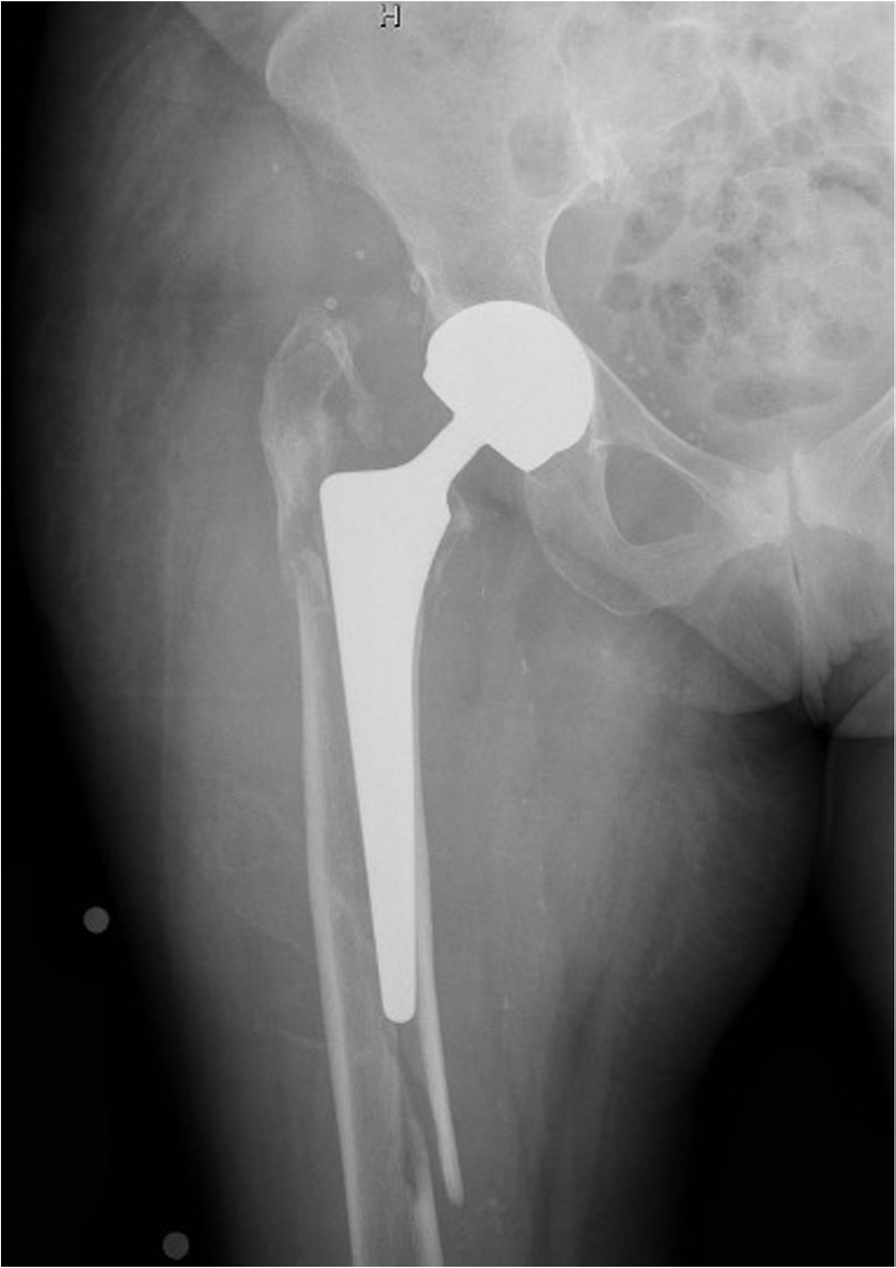
Periprosthetic femoral fracture with stem mobilization and inadequate bone stock

One of the two surgical procedures was performed, based on the type of fracture, the quality of the bone stock, the general conditions of the patient before the fracture (through adequate functional and cognitive assessments) and the associated comorbidities. (Fig. 2). The surgical treatment was mainly decided based on the fracture pattern and stem’s stability, assessed both on preoperative X-Rays and in the operating room, according to the UCS classification. However, the patient’s comorbidities and anesthesiologic consultancy have contributed to clinical and surgical decision. These data have been collected in CIRS score, Oxford and Barthel preoperative score.

No A type fracture have been operated [15]. When the prosthetic implants were considered stable (UCS Classification type B1 or C) [16, 17] ORIF was performed to allow rapid rehabilitation and clinical recovery. In few cases minimally invasive reduction and fixation was performed in patients with very low functional request to reduce surgical morbidity according to preoperative assessment. Prosthetic revision was used in case of loosening of prosthesis stability (type B2) [18] inadequate bone stock (type B3) [19]. We used the postero-lateral approach to the hip with lateral extension to proximal femur, because in our experience it allows a good exposure and direct reduction of the fracture pattern. We performed MIPO approaches only in patients with a C type of fracture, according to classification.

Rehabilitation protocol in both groups focused on rapid recovery of hip mobilization and early muscle strengthening. In RA cases we follow the standard protocol for revision procedures [13], with protection of forced abduction, internal rotation and flexion. Supported weight bearing was allowed for ORIF patients depending on collaboration of the patients, while immediate weight bearing as tolerated was allowed to RA patients.

Authors computed descriptive statistics, looking at medians and interquartile ranges (IQR) for continuous variables and frequencies and proportions for categorical variables. Continuous variables were compared across the two groups using the Student Independent T- test or the Mann-Whitney U test based on their normal or non-normal distribution, respectively. Normality of variables’ distribution was tested by the Kolmogorov-Smirnov test. Categorical variables were tested with the Chi-square and Kruskal-Wallis tests. Differences between pre- and post-treatment variables were assessed using paired T test. Authors used univariate and multivariate ANOVA (MANOVA) with post hoc analyses to compare clinical and surgical variables between groups. Multivariate analysis was performed to explore predictors of Δ Oxford, after adjusting for age, preoperative Barthel and CIRS score. Statistical significance was set at 5%. All tests were two-sided. Analyses were carried out using SPSS v. 24 (IBM SPSS Statistics for Mac, Armonk, NY, IBM Corp).

## Results

The mean age of patients at the time of fracture was 88 years (IQR, 83–96 years) and the median age 90 years (SD ±10.21), 55 patients were female (78.6%) and 15 males (21.4%). In 41 cases left side was affected (58.6%) and in 29 was the right side (41.4%). The median Oxford Score Pre-Surgery was 36 (IQR 22–43, SD + -12.21) and the median Barthel Pre-Surgery score was 87.5 (IQR, 65–100; SD + -22.35). The median comorbidity score of the series, evaluated with CIRS score, was eight (IQR, 5–11; SD + - 4.35). Pre-surgery data are reported in Table 1 and in Supplementary Material.

**Table 1:** Pre-operative data of patients

**Preoperative Features**	
Gender, n. (%)	
Male	15 (21,4%)
Female	55 (78,6%)
Age, median (IQR)	90 (83–96)
Side, n. (%)Side, n. (%)	
Right	29 (41,4%)
Left	41 (58,6%)
Oxford Score Pre-Surgery, median (IQR)	36 (22–43)
Barthel Score Pre- Surgery, median (IQR)	87,5 (65–100)
CIRS Scale, median (IQR)	8 (5–11)
Duncan Classification UCS, n. (%)	
B1 (%)	13 (18,6%)
B2 (%)	32 (45,7%)
B3 (%)	3 (4,3%)
C (%)	22 (31,4%)

**Fig. 3:**
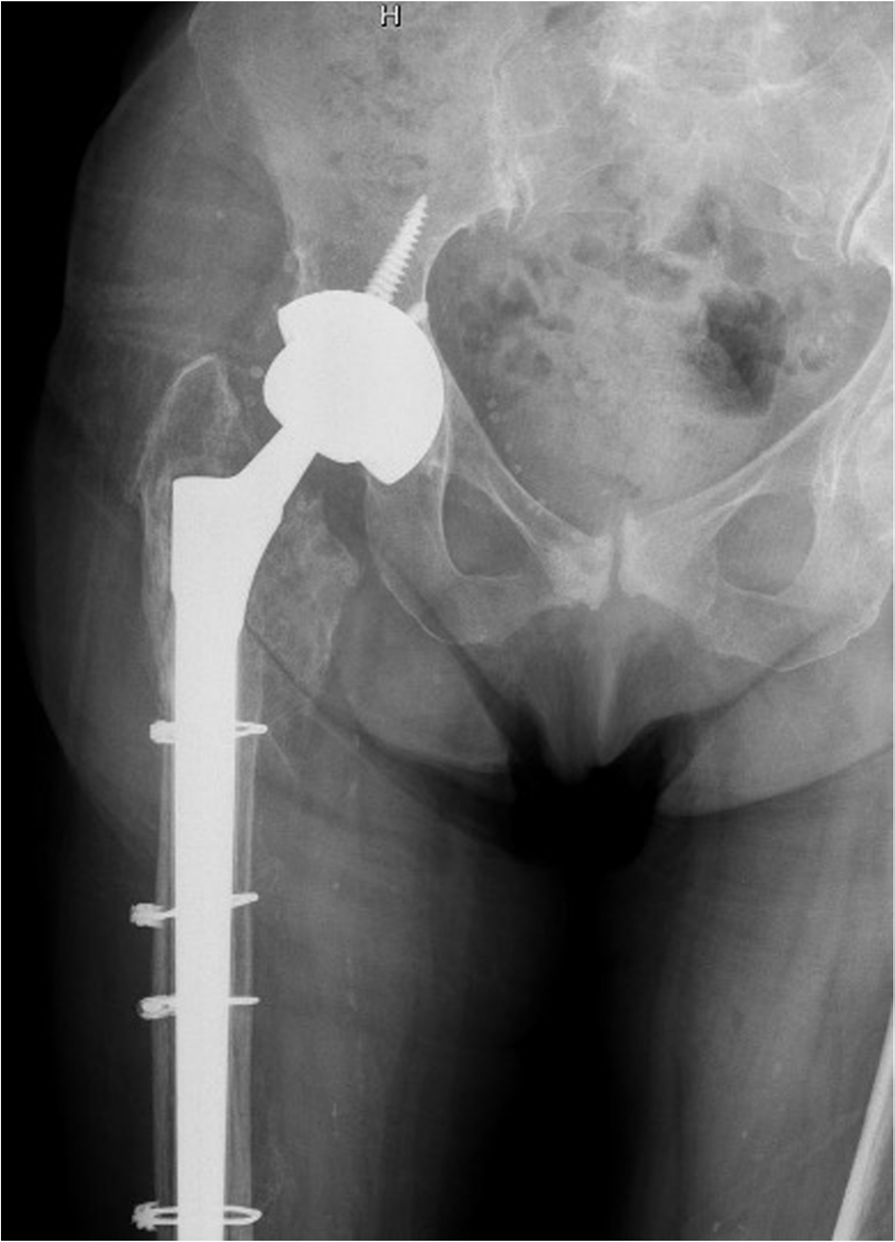
Periprosthetic femoral fracture treated with revision arthroplasty and cerclages

**Fig. 4:**
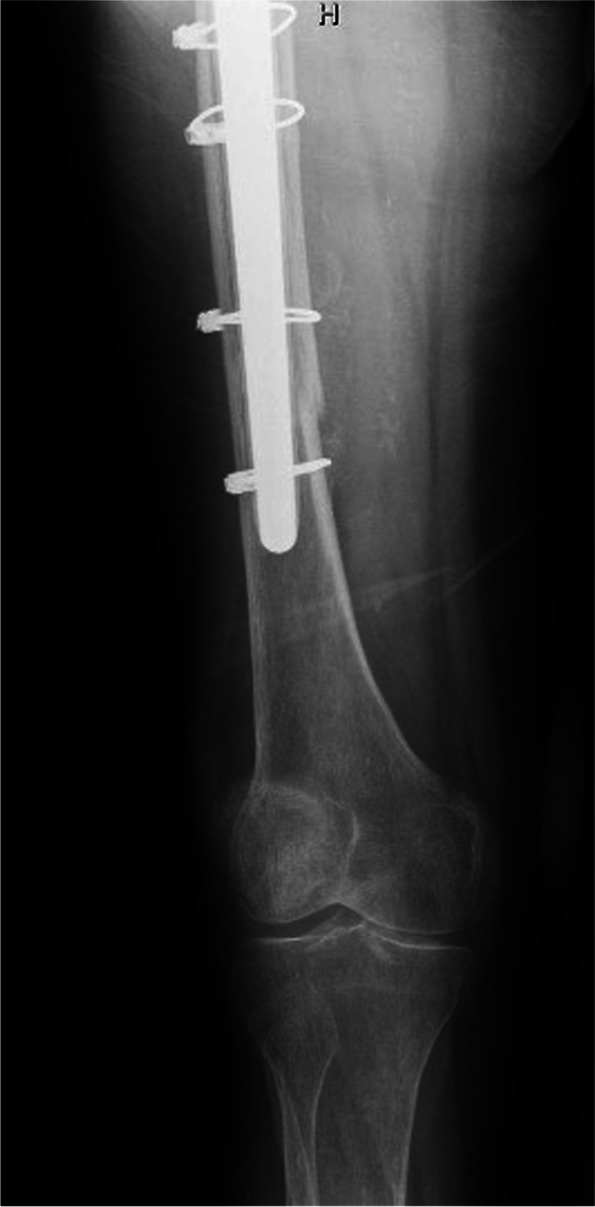
Distal details of revision arthroplasty

According to UCS classification, the most frequent type of fracture was B2 (32 cases, 45.7%) followed by C (22 cases, 31.4%), B1 (13 cases, 18.6%) and B3 (3 cases, 4.3%). (Table 1.) Patients were divided into two groups based on the surgical treatment: G1 treated with ORIF and G2 treated with RA (Fig. 3) (Fig. 4). Of the 45 G1 patients (64.3%), 40 were treated with ORIF with Non-Contact Bridging Periprosthetic Femur Plate System (NCB → PFP, Zimmer) and 3 with the Less Invasive Stabilization System plates (LISS, Synthes). In two cases an implant revision Wagner SL Revision→ Hip Stem (Zimmer) plus ORIF was performed. Given their small number and the similar post-operative protocol, these two cases are included in G1. G2 patients (25 cases, 35.7%) had RA with revision Wagner SL → prosthetic stem.

In 72.9% of total cases (51 cases) Cerclage wires were added to achieve adequate reduction prior to ORIF or RA. Operative procedures are detailed in Table 2. In five patients with B2 type of fractures comorbidities and functional requests have shifted indications from RA to ORIF. These patients had an average age of 98 years, average CIRS score of 10 and all of them have had severe postoperative complications (2 heart failures, 2 pneumonia and 1 infection). The average time between the fracture and the surgery was 4 days (SD + - 2).

**Table 2:** Operative and Post-operative data of patients

**Peri- operative features**	
Type of Surgery, n. (%)	
Reduction and Synthesis (%)	45 (64,3%)
Prosthetic revision (%)	25 (35,7%)
Cerclages, n. (%)	
Yes (%)	51 (72,9%)
No (%)	19 (27,1%)
**Post- operative features**	
Post-operative complications, n. (%)	
Absent (%)	39 (60,9%)
Deep Venous Thrombosis (%)	7 (10,9%)
Aseptic loosening (%)	3 (4,7%)
Dislocations (%)	1 (1,6%)
Pneumonia (%)	5 (7,8%)
Vascular injury (%)	7 (10,9%)
Infection (%)	2 (3,1%)
Blood Transfusion, n. (%)	
Yes (%)	40 (62,5%)
No (%)	24 (37,5%)
Oxford Score Post-Surgery, median (IQR)	32 (19,75 – 40,25)
Barthel Score Post-Surgery, median (IQR)	77,50 (46,25–90)

During hospitalization 40 patients (62.5%) required blood transfusions due to post-surgery anemia. In seven cases (10.9%) post-surgery venous ultrasound (VUS) based on B-mode, combined with color-Doppler US and power imaging techniques and performed on the fifth post-operative day, recorded the presence of a deep vein thrombosis of the lower limb. This was the most frequent post-operative complication, along with seven cases of congestive heart failure (10.9%) and five cases of pneumonia (7.8%), which treated with intravenous antibiotics. Moreover, three patients (4.7%) had implant aseptic loosening, two (3.1%) had an implant prosthetic infection and one patient (1.6%) had a THA posterior dislocation during the follow up. These six patients required an additional treatment and implant revision, equally divided in G1 and G2 group. Post-operative data are reported in Table 2.

The median Oxford Post-Surgery Score was 32 (IQR, 19–40; SD + - 12.07) and the median Barthel Post Surgery Score was 77.5 (IQR, 46.25–90; SD + -26.6). (Table 2).

Results of the univariate analyses between pre-operative, operative and post-operative data in relation to the type of treatment (reduction and synthesis or prosthetic revision) are reported in the Table 3. Pre-operative, operative and post-operative features, like gender (*p* = 0.474), side (*p* = 0.162), blood transfusion (*p* = 0.538) and use of cerclages (*p* = 0.717) were not significantly associated with the type of treatment. The incidence of post-operative complications, deep vein thrombosis, vascular damage, non-unions or consolidation delay and infections, were not statistically significantly different between the two treatments performed (*p* = 0.936), ORIF vs. RA (Table 3). Nominal univariate analyses resulted in significant differences between the two groups in the post-operative and pre-operative Oxford Hip Score (*p* = 0.008) and the CIRS score (*p* = 0.048). Other univariate analysis did not give statistically significant results (Table 3). Finally, the relationship between the type of treatment and the type of fracture was statistically significant (*p* = 0.0001).

**Table 3:** Univariable statistical analyses on patients’ data

	Reduction and Synthesis	Prosthetic Revision	***p*** value
**Pre -operative Features**			
**• Gender, n**			
Female	34	21	0.474
Male	5	10	
**• Side, n**			
Right	15	14	0.162
Left	30	11	
**Pre-operative Oxford, median (IQR)**	36 (24–42.75)	34.5 (22–42.5)	0.980
**Pre-operative Barthel, median (IQR)**	87.5 (67.5–100)	85 (67.5–100)	0.783
**CIRS scale, median (IQR)**	8 (5–11)	6 (4.5–9.5)	0.136
**Peri-operative and Post- operative Features**			
• **Cerclages, n.**			
Yes	33	18	0.717
No	13	7	
• **Blood Transfusion, n.**			
Yes	27	13	0.538
No	13	8	
Present	19	2	
• **Post-operative complications**			
Yes	20	13	0.936
No	25	12	
• **Post-operative and Pre-operative Oxford (Δ Oxford)**			**0.008**
• **Duncan Classification**			**0.0001**
**Post- Operative and Pre-operative Oxford Hip score**			
**CIRS SCORE**			**0.048**

MANOVA with post hoc analysis showed a significant difference according to Δ Oxford between patients undergoing reduction and synthesis or prosthesis revision (*p* = 0.042).

At multivariable analysis, after adjusting for age, preoperative Barthel and type of fracture assessed by UCS classification, the CIRS score was independently associated with Oxford Score improvement after surgery (*p* = 0.024).

## Discussion

Treatment of periprosthetic femoral fractures (PPFx) often require senior surgeons with competence both in ORIF and RA. Further, the patients themselves are challenging as they usually present severe comorbidities [20]. The treatment of PPFx has been associated with high risk of failure, poor outcomes and worst survival outcome compared to the general population 1 year after surgery. This still applies 5-8 years after the surgery [10, 21].

The goal of the treatment is to restore stem stability and limb alignment, recover pre-fracture functional mobility and allow early mobilization. In B1 and C fracture types, the standard treatment is ORIF, but in older patients it could be demanding due to weakened bone quality and potential bone loss [8, 22]. The recommended treatment for B2 and B3 fractures is femoral stem revision, which may be reinforced with plating or isolated cerclage wires, using long stems with diaphyseal fixation to achieve a more stable construct or revision cemented stems [1, 23].

In this study, we found a strong statistical correlation between the type of fracture, classified using the UCS classification, and the surgical treatment used, according to classification. Results of this retrospective study confirm validity of UCS classification system to guide clinical decision-making for treatment of periprosthetic hip fractures.

We evaluated the clinical outcome based on the difference in Oxford Score post and pre-surgery, comparing RA and ORIF. The univariate analysis showed a better clinical post-operative outcome for patients treated with prosthetic revision (B2) compared to those treated with ORIF (B1 or C). These findings are not completely aligned with literature, which does not find different functional outcomes depending on treatment [24]. To further understand this difference, we compared functional outcomes of ORIF B1 fractures to ORIF C fractures and found no significant differences between the two (*p* = 0.143). Therefore, we can conclude that the better outcomes associated with RA are not related to the fact that ORIF group had both B1 and C fractures, but instead it may be associated to better functional postoperative status of patients treated with revision stems. This highlights how revision, when appropriate, can obtain better outcomes in elderly patients. These results seem to prompt surgeons to prefer stem’s revision, especially when they are undecided about the best surgical treatment for the patient [25].

Although functional outcomes are closely related to the type of surgical treatment, the multivariate analysis reveals that they are also strongly influenced by the patients’ age, pre-operative Barthel Score and the CIRS score.

The clinical outcome, expressed as the Delta Oxford Score (post- and pre-operative), is positively correlated to the CIRS score. The CIRS score captures several comorbidities, including some that have little impact on the post-surgery rehabilitation and functional outcome of PPFx, such as gastrointestinal disorders, and others that are extremely important in PPFx, like respiratory diseases. Since each comorbidity is weighted equally, patient with the same CIRS score may have very different risk. Indeed, we recorded different Delta Oxford Score in patients with the same CIRS score. The association between CIRS score and Delta Oxford score is also related to the age, pre-operative Barthel score and type of fracture, but does not correlate with the surgical treatment. It is important that patients be evaluated by a multidisciplinary team, to consider not only the surgical performance but also each patient’s comorbidities, compliance and adherence to rehabilitation pathway and risk of post-operative complications.

In the literature, survival after PPFx is worse than after any other cause of revision THA, such as infection, aseptic loosening, and dislocation. Cnudde et al. [10] found that survival in the repeated surgery after THA is influenced by the reason of re-operation, and that PPFx patients have a five-year survival rate of 54%, worse than the rates for RA with different complications [26]. Young et al. [27] analyzed the functional outcome of revision for periprosthetic fractures and found that patients had poorer outcome and higher death rates compared to those undergoing revision THA for aseptic loosening. Similarly, Young underlines that patients who had been operated by experienced surgeons and at larger centers have lower mortality rate.

According to the literature, patient mortality rates at 1 year remains high (13–17%) despite improvements in surgical and fixation techniques [28, 29]. However, Bhattacharyya et al. [30] reported that 1 year mortality rate in patients treated for type B fractures with internal fixation was 33% while those treated with femoral stem revision experienced a 12% mortality rate. Tucker et al. reported that Vancouver classification is an accurate system to choose the surgical treatment, but it is not correlated with the one-year mortality rate, nor to the length of the surgical procedure [31].

Stoffel et al. [25] compared the functional outcomes of patients undergoing ORIF or RA in Vancouver type B2 and B3 PPFx. Their study highlighted that ORIF could be a viable treatment option considering the type of prosthetic stem, anatomical reduction of fracture and intact cement mantle. Limit of the present study is due to data collected from a single department, even if with large numbers of proximal femoral fractures and prosthetic implants and that it is a retrospective study with lack of randomization. In addition, we did not analyse the differences between cemented and uncemented stems as we had only six cases, and further investigation is needed to see if there are significant difference between two group.

## Conclusions

We reported a strong correlation between the type of fractures and the surgical treatment used, according to the UCS classification. This credits the use of UCS classification to delineate surgical treatment. Both ORIF and RA, if done with correct indication, may afford good functional and clinical results. However, we found a better functional result of RA patients and these results are influenced, like in proximal femoral fracture patients, from the patients’ age, Barthel index and CIRS score. The relation, even if not linear, between Δ Oxford and CIRS Score highlights the importance to adapt surgical treatment to patients’ conditions and confirm that good results can be expected with rapid recovery.

## Data Availability

The datasets generated and/or analyzed during the current study are not publicly available due but are available from the corresponding author on reasonable request.

## Abbreviations

THA: Periprosthetic femoral fractures in Total Hip Arthroplasty
UCS: Unified Classification System
CIRS: Cumulative illness rating scale
PPFx: Periprosthetic femoral fractures
ORIF: Open reduction and internal fixation
RA: Revision arthroplasty
IQR: Interquartile ranges
